# Forty years of monitoring increasing sea turtle relative abundance in the Gulf of Mexico

**DOI:** 10.1038/s41598-023-43651-4

**Published:** 2023-10-11

**Authors:** Jacob Andrew Lasala, Melissa C. Macksey, Kristen T. Mazzarella, Kevan L. Main, Jerris J. Foote, Anton D. Tucker

**Affiliations:** 1 Sea Turtle Conservation and Research Program, Mote Marine Laboratory, 1600 Ken Thompson Parkway, Sarasota, FL 34236 USA; 2 Parks, Recreation and Natural Resources, Sarasota County, 1660 Ringling Boulevard, Sarasota, FL 34236 USA; 3 Present Address: Marine Science Program, Biodiversity and Conservation Science, Department of Biodiversity, Conservation and Attractions, Kensington, WA Australia

**Keywords:** Biogeography, Herpetology

## Abstract

Longitudinal data sets for population abundance are essential for studies of imperiled organisms with long life spans or migratory movements, such as marine turtles. Population status trends are crucial for conservation managers to assess recovery effectiveness. A direct assessment of population growth is the enumeration of nesting numbers and quantifying nesting attempts (successful nests/unsuccessful attempts) and emergence success (number of hatchlings leaving the nest) because of the substantial annual variations due to nest placement, predation, and storm activity. We documented over 133,000 sea turtle crawls for 50.9 km of Florida Gulf of Mexico coastline from 1982 to 2021 for a large loggerhead turtle nesting aggregation and a recovering remnant population of green sea turtles. Over time both species have emerged to nest significantly earlier in the year and green sea turtle nesting seasons have extended. Nest counts and hatchling production for both species have significantly increased, but the rate of emergence success of hatchlings leaving nests has not changed for loggerheads and has declined for green sea turtles. Sea level rise and coastal developments undoubtedly influence coastal habitats in the long-term, impacting nest site selection and potential recruitment from the loss of emerged hatchlings. However, the present indications for steady Gulf of Mexico recovery of loggerhead and green sea turtles counter findings of the Florida Atlantic coasts. This study indicates that effective conservation practices can be detected within time scales of 1–2 turtle generations.

## Introduction

Long term data sets and analyses are essential to conservation plans of a species^1–3^. In ideal situations, observations of behavior can be combined with census counts of individuals^4,5^ thus allowing researchers to quantify how populations change over time. For many species, especially those that are threatened or endangered, these changes may be difficult to observe^6,7^ and proxies for direct observation must be used instead. This is especially true for animals that migrate from foraging grounds to breeding grounds^8–10^ and might not be easily accessible year-round, such as marine turtles.

Marine turtles are one of the few reptiles that migrate long-distances between foraging areas and breeding areas^11,12^. Observational data linked with mark-recapture histories indicate that migration cycles are resource driven and food availability can affect the time-frame between breeding seasons^13–15^. Remigration cycle shifts may influence recruitment rates, possibly impacting a population’s growth over time. Some demographic studies of marine turtles occur in-water through mark-recapture techniques^16–18^, but most estimates of population size are made during the breeding season from evidence of female tracks at nesting beaches. Females, their nests, and the resulting hatchlings are accessible on the beach during the nesting season and provide a baseline for population trends^19–21^. Marine turtles reach sexual maturity later than other reptiles and have a long lifespan^22–25^ reducing the likelihood that changes within a population are immediately apparent. Long term beach monitoring data can provide a reference point for the health of that breeding population^26–28^ and by coupling nest counts with reproductive success data researchers can predict future issues stemming from population fitness changes^29^.

The prevailing theory of marine turtle population structure suggests that data from one beach may not be enough to see the full picture of a breeding population^30^. Marine turtle genetic population structure is driven by female natal philopatry^31^ and gene flow occurs throughout the region as males travel along the coast toward their breeding areas^32,33^. More recent studies propose that population subdivision creates pockets of subpopulations^30,34,35^. It is therefore critical to document monitoring for more than a decade of contiguous nesting beaches to assess a region’s population trend^36,37^.

Marine turtles are threatened globally. In some regions they are hunted for their meat and shells, their eggs are poached, and turtles can be caught as bycatch^38–40^. Pollution in the ocean increases the likelihood of plastic ingestion or death and boat strikes are prevalent near populated areas^41,42^. Nesting beaches are at risk from armoring/sand nourishment activities, their nests are at risk of predation, and hatchling disorientation or misorientation from artificial lighting^43–45^. However, relative abundance research provides conservation managers with tools to better protect these animals. The loggerhead sea turtle (*Caretta caretta)* nesting aggregation on the Northwest Atlantic Ocean is the largest in the world^46^, and 90% of these clutches are laid in Florida^47,48^. Green sea turtle (*Chelonia mydas*) nesting populations have been rapidly increasing in Florida since 1998^49,50^; these increases have resulted in the down-listing of this species from “endangered” to “threatened”^51^. Much of the research on these and other species of marine turtles occurs on the East coast of Florida, where nesting density is high^52–54^. Few studies have focused on Florida’s Gulf of Mexico coastline^28,55,56^, despite the growing nesting populations in this region. Programs that have monitored marine turtle nesting over time are invaluable to identifying trends of how populations have changed through time^27,57,58^.

The primary goal of this study was to determine how nesting counts and hatchling production have changed over time and to quantify how nesting turtles on these beaches are contributing to the greater Gulf of Mexico marine turtle populations. The secondary goal was to assess how the different beaches contribute to the population and to identify regions that are more or less productive to inform regional conservation managers of these trends.

## Methods

### Study sites

Since 1982, the Mote Marine Laboratory Sea Turtle Conservation and Research Program (MML STCRP) has standardized annual monitoring for 50.9 km of nesting habitat across six beaches in Sarasota County, Florida. With 40+ years of continuous data, STCRP is uniquely placed to identify trends in the growing population and to estimate how this population’s breeding behavior has changed over time. Sarasota County hosts the largest nesting aggregation of loggerheads within the Gulf of Mexico and is an excellent representation of the growth of nesting in the region^59,60^.

**Figure 1:**
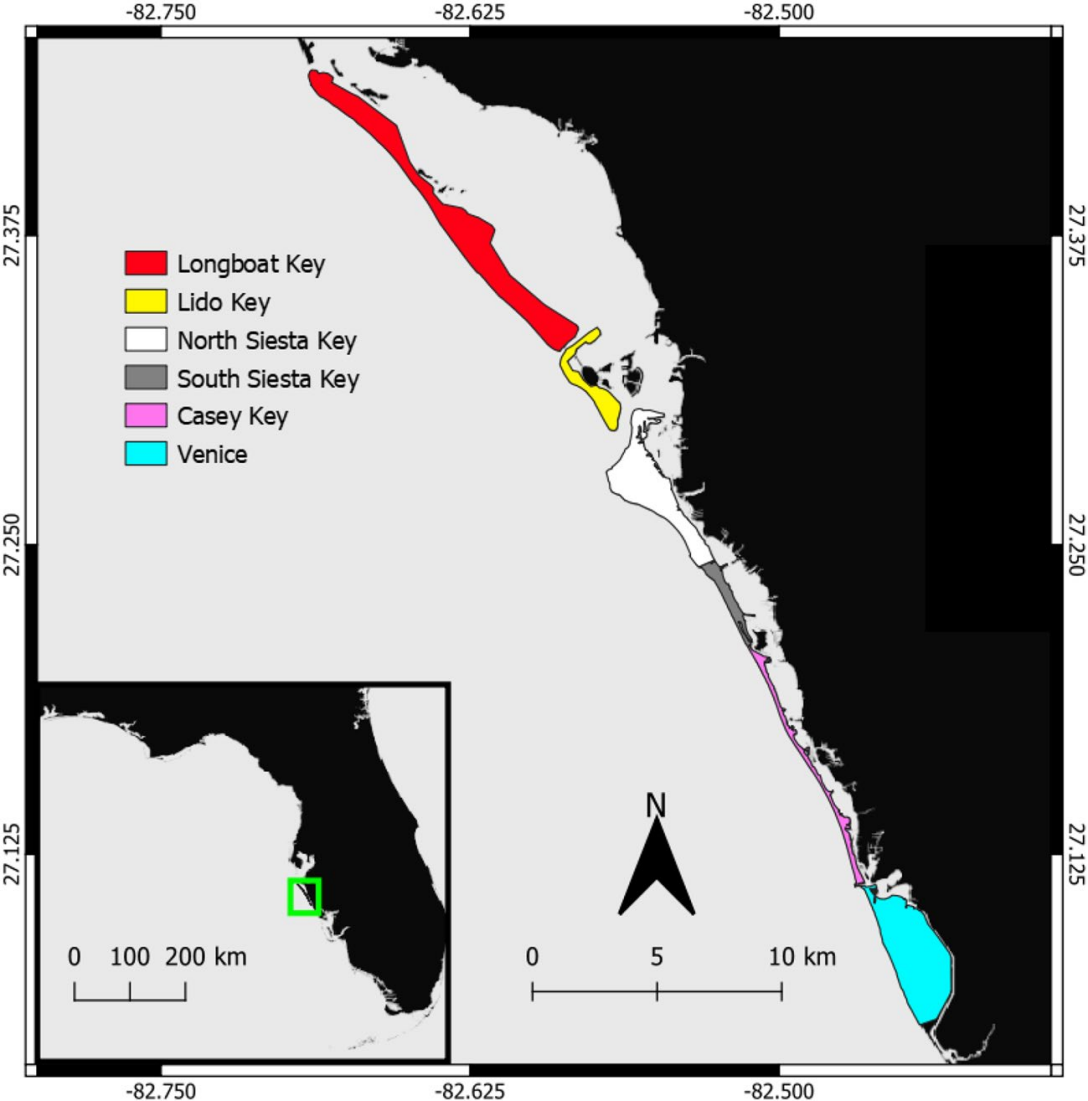
Nesting beaches monitored by MML STCRP in Sarasota Florida. North to South they are Longboat Key, Lido Key, Siesta Key (North and South), Casey Key and Venice. Beaches vary in physical parameters (sand type, nourishment, obstructions, armoring) and these differences are highlighted in Table 1.

During the nesting season (April–October), MML STCRP staff and volunteers monitor sea turtle nesting during morning patrols on six beaches in Sarasota County. The majority of these barrier islands are considered to be critically eroded^61^, threatening suitable nesting habitat and residential property. Residents of these coastal communities have attempted to safeguard their beaches through beach nourishment and/or implementing coastal armoring techniques. These activities can negatively impact sea turtle nesting behavior^62–65^. Each beach varies in length, sand color and sand grain, armoring, nourishment, and profile (Table 1, Fig. 1).

**Table 1:** Six beaches in Sarasota County, Florida, USA that STCRP monitors annually (mapped in Fig. 1). Each beach is unique in length, sand color/albedo/grain size, beach profile, nourishment, and armoring methods.

Beach	Length km (mi)	Sand color	Sand grain	Beach profile	Nourished? (# of times)	Armoring
Longboat Key	17.1 (10.6)	White–brown	Fine–medium	Moderate	Yes (18)	Seawalls, groins, rock revetments
Lido Key	5.3 (3.3)	White	Fine	Flat–moderate	Yes (10)	Groins, rock revetments
North Siesta Key	5.2 (3.2)	White–brown	Very fine	Flat	No	Outfalls, groins, rock revetments
South Siesta Key	4.1 (2.5)	Dark	Coarse	Dynamic	Yes (2)	Rock revetments
Casey Key	11.8 (7.3)	Brown	Medium	Flat–moderate	No	Seawalls, groins, rock revetments, pilings, jetty, sandbags, stepped revetments, geotextile container
Venice	7.4 (4.6)	Dark	Coarse	Dynamic	Yes (5)	Outfalls, rock revetments, jetty

### Coordinated citizen science nesting surveys

All beaches monitored by MML STCRP fall under the Statewide Nesting Beach Survey (SNBS) program under the Florida Fish and Wildlife Conservation Commission (FWC). Siesta Key is also part of a standardized data collection program called the Index Nesting Beach Survey (INBS). These two programs are designed to assess the distribution and relative abundance of sea turtle nesting across Florida to inform beach managers of coastal development issues and promote the recovery of sea turtle populations^57,66^. Survey data are collected through permit holders, but the core of the data could not be collected without the help of trained volunteers who patrol the beaches to identify and document sea turtle ocean emergences. One such volunteer group: Longboat Key Turtle Watch (LBKTW) started quantifying nesting activity in 1969 and was a pioneering example for other long-term projects in the region. Since 2005, volunteers log an average of 19,000+ hours every nesting season.

Annual daily beach monitoring was conducted from April 15 (May 1st prior to 2016) through October 31, or 3 days after the final hatchling emergence. Permitted personnel traveled the shoreline at dawn above the mean high-water line to document turtle nesting activity. Using Florida Fish and Wildlife’s marine turtle guidelines^67^, each activity was classified as a nest or a non-nesting emergence (also known as a false crawl) and the species was identified via crawl pattern. Nest counts were defined as the number of crawls that emerged from the ocean that resulted in the laying of a clutch of eggs within the sand before the turtle returned to the ocean. A non-nesting emergence was defined as an emergence that did not result in egg deposition. *Nesting success* was calculated as the proportion of emergences that resulted in a nest in relation to the total number of crawls and served as an indicator of a beach’s nesting suitability^68–72^:

1 $$Nesting\,Success= \frac{\# nests}{\# nests+\# false\,crawls}$$

*Nesting density* is an index of nest distribution and is used as a metric for conservation success when assessed over time^73,74^:

2 $$Nesting\,Density= \frac{\# nests}{distance\,(km)}$$

Most clutches were left in situ*,* but clutches laid during active nourishment projects were relocated by 9 a.m. the morning after deposition—eggs were transported to a location higher on the beach and placed into an artificially built nest chamber that closely resembled the original nest chamber in shape, size, and depth. Additionally, regulations reflecting the potential negative impacts of nest relocations were not set prior to 1987 and many nests were relocated to protective “hatcheries”.

Turtle crawl locations were documented in relation to the nearest Florida Department of Environmental Protection (FDEP) range monument to the north and, beginning in 2004, with global positioning system (GPS). Starting in 1997, personnel measured the distance from the nest or false crawl apex to the closest upland vegetation/barrier and from the nest or false crawl apex to the day’s mean high-water line. By adding these two values together, total *beach width* was estimated. These measurements were used to assess nest site selection in relation to the lower, middle, or upper thirds of the beach, regardless of beach width.

All nests prior to 2013 were monitored: eggs in the clutch were verified, nest sites were marked with stakes, and nest sites were checked daily for depredation, disorientation/misorientation, wash-overs/inundation, wash outs and hatchling emergences. By 2013, the nesting population had grown too large to monitor and stake every nest. Following communication with FWC, MML STCRP shifted to a new sampling protocol based on turtle species and beach nesting density. On moderate to high density non-nourished beaches all loggerhead clutches laid on Wednesdays were verified and thereafter monitored; all nests on low density beaches, all rare species [green, Kemp’s ridley (*Lepidochelys kempii*), leatherback (*Dermochelys coriacea*), and potential hybrid] nests, research nests, relocated nests, and all nests in nourishment areas were monitored.

Nest depredation occurred when a native or non-native organism preyed on incubating sea turtle eggs and hatchlings. In Sarasota County, depredation occurs from native predators (raccoons, armadillos, ghost crabs, coyotes) and from non-native predators (fire ants). Depredation events included predators digging into a nest and removing or damaging nest contents, subsequently impacting hatchling production. From 1982 to 2020, to mitigate depredation, self-releasing cages and/or screens were installed at all verified rare species nest sites and some loggerhead nest sites in high depredation areas.

After a monitored nest hatched and the hatchlings emerged, MML STCRP determined whether the hatchlings disoriented/misoriented en route to the Gulf. If a hatchling is disoriented, they will wander on the beach without direction (typically from skyglow), and if a hatchling is misoriented they typically travel to an artificial source of light and not the ocean^75^. Disorientation or misorientation events can be caused by natural and artificial lighting^76^ and can have an impact on hatchling survival^77^. MML STCRP documents every disorientation/misorientation event, but their impact on hatchling survival is not fully described here.

*Incubation duration* was assessed by subtracting the date hatchlings emerged from the nest by the date the nest was laid. MML STCRP staff excavated the nest three days following an observed emergence. If an emergence was not noted, excavations occurred after 70 days of incubation (80 days on North Siesta Key - where cooler sand temperature extends the incubation duration, and for all leatherback nests). During an excavation, nest contents were classified by counting hatched eggshells, hatchlings, and remaining unhatched eggs^78^. This method has not changed over the course of the study reducing error^58^. These counts were used to determine the emergence success of the nest. *Emergence success* quantified how many hatchlings exited the nest chamber and made it to the surface:

3 $$Emergence\,Success= \frac{\# hatched\,eggs-(live\,hatchlings\,in\,nest\,chamber+dead\,hatchlings\,in\,nest\,chamber)}{total\,clutch\,size}$$

Emergence success was calculated for all nests from 1982 to 2012, but only for monitored nests from 2013 to 2021 (see above). To remove potential bias from relocations, only in situ nests were included in the emergence success calculations. Partial depredations and partial washouts were removed from overall success calculations because we could not ascertain how many eggs were lost. Full depredations and full washouts were included as zero (0) success because the entire clutch was removed from productivity. By compiling these data, the *annual hatchling production* was assessed using regression analysis by beach and year. Two values are provided here: (1) a minimum number of hatchlings confirmed from all excavations from 1982 to 2021 and (2) a higher estimated number of hatchlings if MML STCRP had monitored and excavated every nest post-2013 (a further explanation can be found in Supplementary Information A)*.*

### Data standardization and analysis

All data were assessed and analyzed between 1982 and 2021. All analyses were performed on loggerhead and green data sets. MML STCRP also identified nests and false crawls from Kemp’s ridleys, leatherbacks and loggerhead/hawksbill hybrids (confirmed by genetics, B. Shamblin *pers comm.*), but the total number of these rare sea turtles nesting in the region remains too low to make inferences about overall nesting behavior. Further, adjustments were made to the beach protocol over time (see above); thus, not all parameters could be compared and analyzed throughout the timeframe. Nest counts, nesting density, nesting success, and incubation duration were all compared for the full 40-year monitoring timeframe (emergence success for 35 years). Nest location placement was only compared for the period 1997–2021. A separate data set removing beaches by the year that they were nourished was analyzed and the results are presented in Supplementary Information B.

All analyses were run in Program R 4.0.3^79^, all figures were made using package *ggplot2*^80^, and all maps were made using QGIS 3.16^81^. All data were tested for normality (Shapiro–Wilk or Kolmogorov–Smirnov tests) and homoscedasticity (Levene’s test). Most data were non-normal and most tests were non-parametric. To identify if there were differences in nest counts, nesting density via beach or year, generalized linear models were assessed and post-hoc Dunn tests were performed (package *dunn.test*)^82^. Siegel nonparametric linear regression (package *mblm*)^83^ was used to determine if first and last nest dates had changed over the course of the study and for analysis of incubation duration and emergence success over time. Both nesting success and emergence success are proportions that range between values of 0 and 1. Modified beta regression models (package *betareg*)^84^ were run to explore whether nesting success or emergence success were affected by abiotic factors including Year, Beach Width, Date of Activity and Latitude (proxied by FDEP monument). Finally, Kruskal–Wallis tests were used to compare nest site selection in relation to the spatial placement on the beach.

## Results

MML STCRP documented 133,957 marine turtle crawls from 1982 to 2021; 48.8% of these crawls resulted in nests (65,321). Of those, 99.0% (64,692) were laid by loggerheads. There were 608 green sea turtle nests, ten Kemp’s ridley nests, five leatherback nests and two known loggerhead/hawksbill hybrid nests.

**Figure 2:**
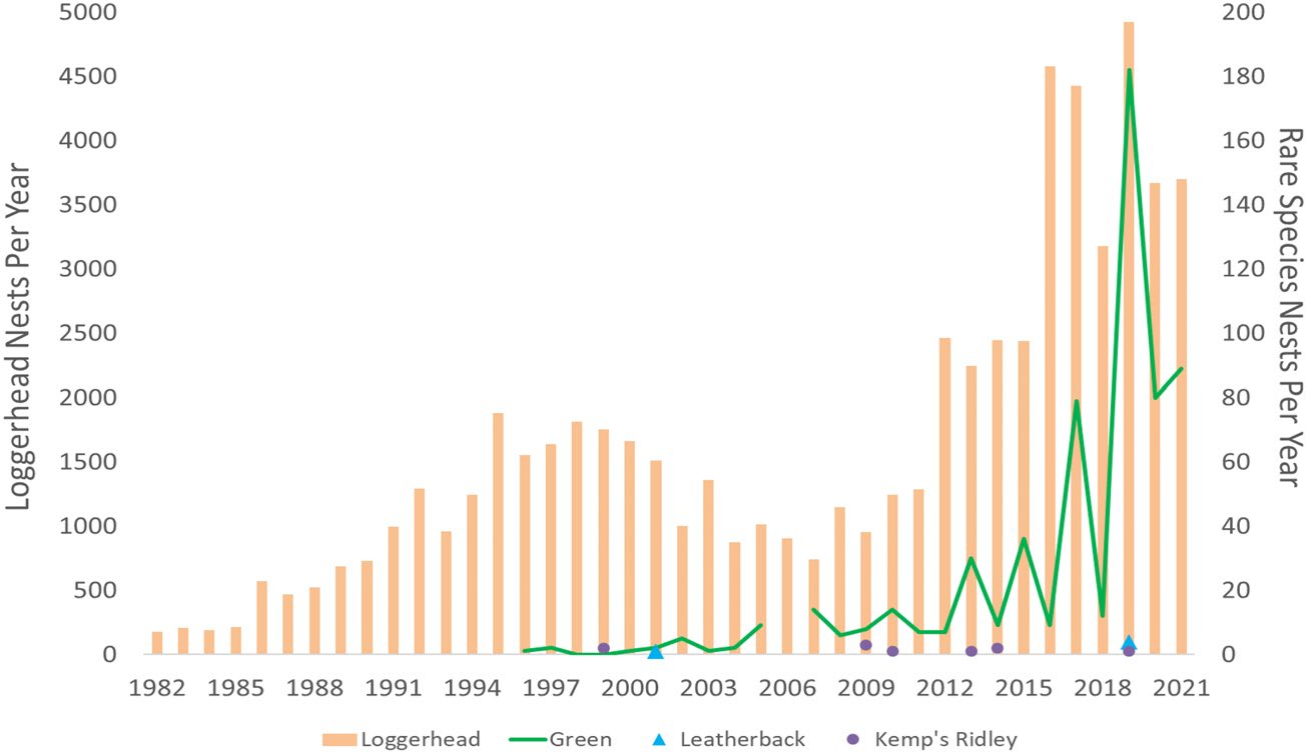
Nest counts for the period 1982–2021 from all beaches monitored by MML STCRP. Rare species include green sea turtles, leatherbacks, and Kemp’s ridley turtles.

Nesting counts for loggerheads and *rare species* have increased since monitoring began (Fig. 2). The nest count data for both loggerheads and green sea turtles were not normally distributed (Shapiro–Wilk: loggerheads: W = 0.71, p < 0.001; green sea turtles: W = 0.42, p < 0.001) and residuals were non-normal for both species. No other species data were analyzed due to small sample size. For loggerheads, there were significant differences in nest counts over time and by beach (χ^2^ = 84.59, df = 39, p < 0.001; χ^2^ = 142.83, df = 5, p < 0.001 respectively). Post-hoc tests revealed that Casey Key had significantly higher nest counts than the other beaches and Lido Key and North Siesta Key had significantly fewer nests than the other beaches. For green sea turtles, even though the nesting population was growing there was no significant difference in nest counts over time (χ^2^ = 28.03, df = 26, p = 0.36). However, green sea turtle nesting counts were significantly higher on Casey Key than on all of the other beaches (χ^2^ = 16.91, df = 5, p < 0.001).

### Species phenology, density, and success

**Figure 3:**
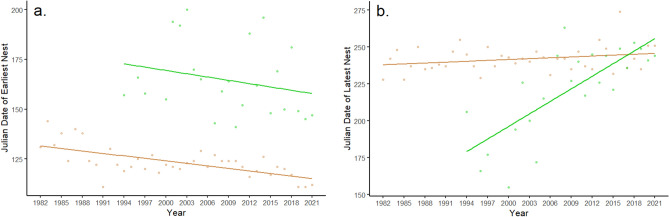
Julian date of earliest (**a**) and latest (**b**) laid nests for loggerheads (tan) and green sea turtles (green) for the period 1982–2021. Both species are significantly laying their nests earlier over the 40-year timeframe and greens are significantly laying their last nests later.

The earliest loggerhead clutch was laid on April 20 and the latest was laid on September 30, and for green sea turtles: May 21 and September 19, respectively. Emergences resulting in a nest have significantly shifted earlier in the year for both loggerheads (V = 37, df = 38, p < 0.001) and for green sea turtles (V = 76.5, df = 22, p = 0.037; Fig. 3). Nesting season has not significantly shifted longer for loggerheads (V = 437, df = 38, p = 0.104), but has shifted significantly later for green sea turtles (V = 298, df = 22, p < 0.001).

**Figure 4:**
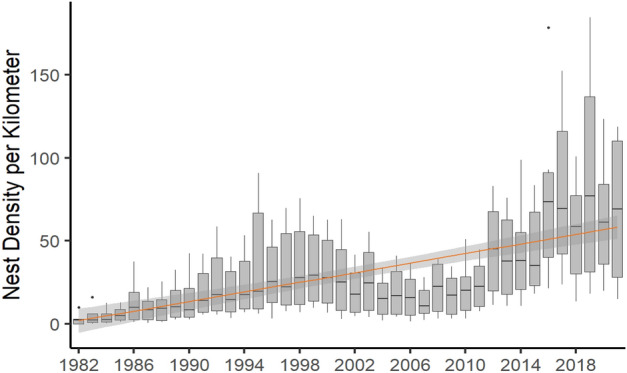
Loggerhead nesting density per km over time for the period 1982–2021. Nesting density significantly increased over time (the grey band represents the 95% confidence interval). Grey dots represent outliers from those years. Southern beaches had higher densities than northern beaches, resulting in high variation between the beaches from year to year.

Nesting density for both loggerheads and green sea turtles were not normally distributed (W = 0.79, p < 0.001; W = 0.49, p < 0.001) and neither were the residuals. For loggerheads, nesting density (x̄: 30.7 nests/km) significantly increased over time and was significantly different between beaches (χ^2^ = 96.12, df = 39, p < 0.001; χ^2^ = 129.54, df = 5, p < 0.001; Fig. 4). Loggerhead nesting density was highest on Casey Key and lowest on North Siesta Key. For green sea turtles, nesting density (x̄: 0.822 nests/km) increased over time—but not significantly (χ^2^ = 30.43, df = 25, p = 0.21); however, there were significant differences by beach (χ^2^ = 16.36, df = 5, p = 0.01). Post-hoc tests determined that green sea turtles prefer to nest on Casey Key and South Siesta Key and have never nested on North Siesta Key.

Nesting success for both species was not normally distributed (loggerheads: W = 0.97, p < 0.001; green sea turtles: W = 0.91, p < 0.001) and neither were their residuals. Loggerhead nesting success declined over time, but not significantly (x̄: 0.49 ± 0.14; χ^2^ = 51.57, df = 39, p = 0.09). Loggerhead nesting success significantly varied between beaches (χ^2^ = 68.39, df = 5, p < 0.001). Nesting success was highest on South Siesta Key (0.57) and lowest on North Siesta Key (0.38). Nesting success for green sea turtles varied widely (x̄: 0.53 ± 0.33), but it did not significantly vary by year (χ^2^ = 30.967, df = 30, p = 0.42), nor by beach (χ^2^ = 4.577, df = 5, p = 0.47), despite the lack of nesting on North Siesta Key.

### Species incubation and emergence success

**Figure 5:**
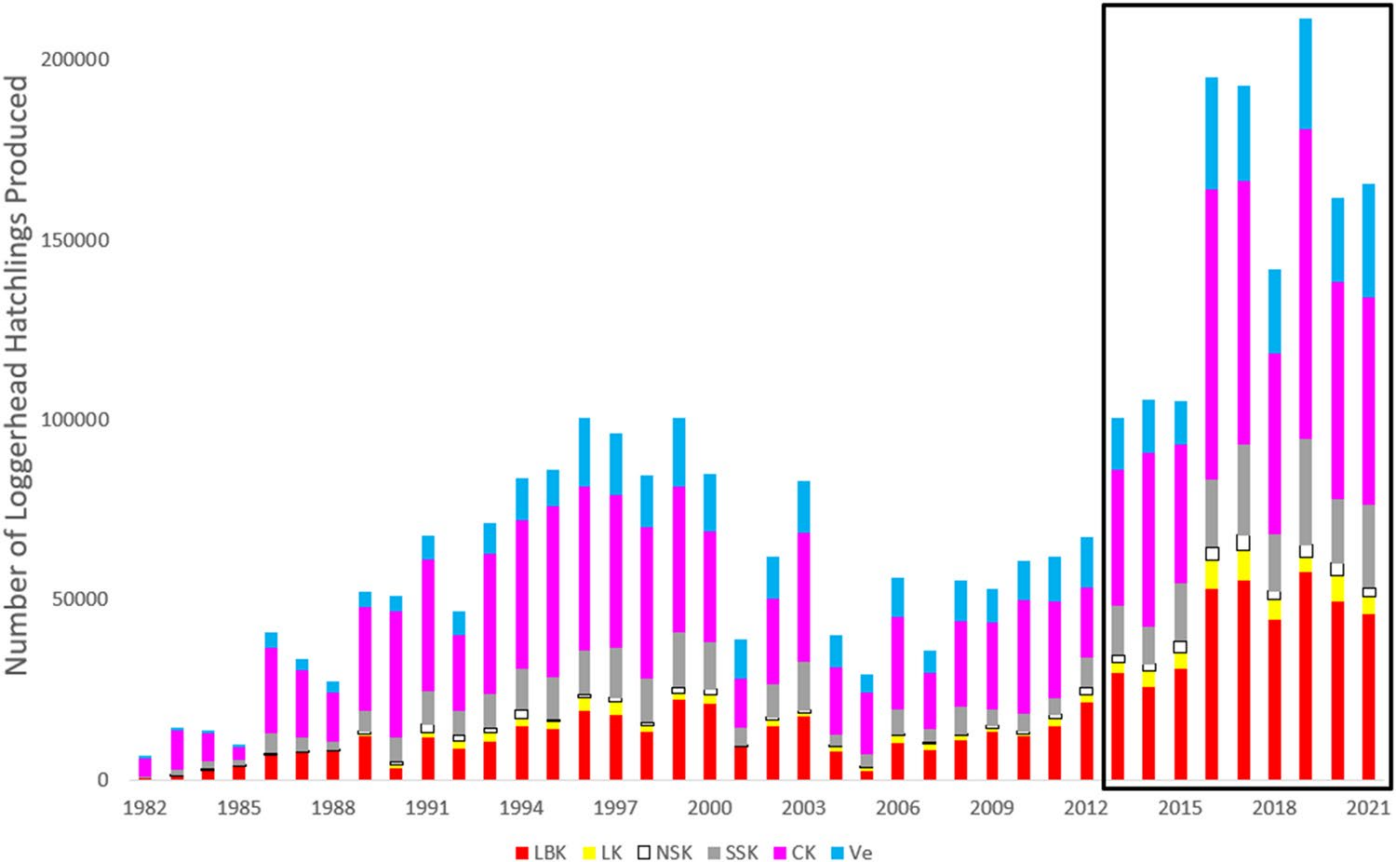
Estimated number of loggerhead hatchlings produced per year. Prior to the black box, all nests were excavated and the hatchling numbers were confirmed. The black box includes estimated number of hatchlings based on regression analysis (Supplementary Information A).

The average incubation duration and emergence success for loggerheads across all beaches was 58.9 days and 52.1%, respectively (a full breakdown by beach is in Supplementary Information). Over the course of the study, neither incubation duration, nor emergence success were normally distributed (Kolmogorov–Smirnov: D = 1, p < 0.001; D = 0.5, p < 0.001 respectively), and neither were their residuals. Loggerhead incubation duration has significantly increased over time (V = 741, df = 37, p < 0.001) and was significantly different between beaches (χ^2^ = 113.39, df = 5, p < 0.001). The longest average incubation duration was on North Siesta Key (77.6 days in 2015) and the shortest average on Venice (50.9 days in 1994). Loggerhead emergence success decreased over time—but not significantly (V = 310, df = 38, p = 0.181), but emergence success did significantly vary between beaches (χ^2^ = 54.50, df = 5, p < 0.001). Casey Key had the highest average emergence success (61.1%) and North Siesta Key the lowest (28.3%). Over the course of the study, the minimum number of loggerhead hatchlings that emerged from these nests was 2,180,700. Using multiple regression models (outlined in Supplementary Information A), a higher value of 3,078,811 is estimated. Casey Key produced the most hatchlings annually (x̄: 24,000–33,500 per year, see Fig. 5).

Hatchlings emerged from 484 of the 608 green sea turtle nests (79.6%), resulting in 40,580 hatchlings. The average incubation duration was 58.4 days, and the average emergence success was 63.7%. Over the course of the study, neither incubation duration nor emergence success were normally distributed (W = 0.954, p < 0.001; W = 0.803, p < 0.001 respectively), nor were their residuals. Incubation duration has significantly increased over the course of the study (V = 226, df = 20, p = 0.001) and incubation duration significantly varied between beaches (χ^2^ = 45.87, df = 4, p < 0.001). Lido Key had a longer average incubation duration than both South Siesta Key and Venice. Green sea turtle emergence success has significantly decreased over time (V = 6, df = 21, p < 0.001). However, there was no significant difference in emergence success between beaches (χ^2^ = 6.69, df = 4, p = 0.15). There were 320 Kemp’s ridley hatchlings that emerged from nests (incubation x̄: 63.8 days, emergence success x̄: 27.3%) and 165 loggerhead/hawksbill hybrid hatchlings (incubation x̄: 54 days, emergence success x̄: 50%). None of the five leatherback nests hatched.

### Effects of nest site selection on nest success

Only statistically significant values are explained below; full model breakdowns are in Supplementary Information.

**Figure 6:**
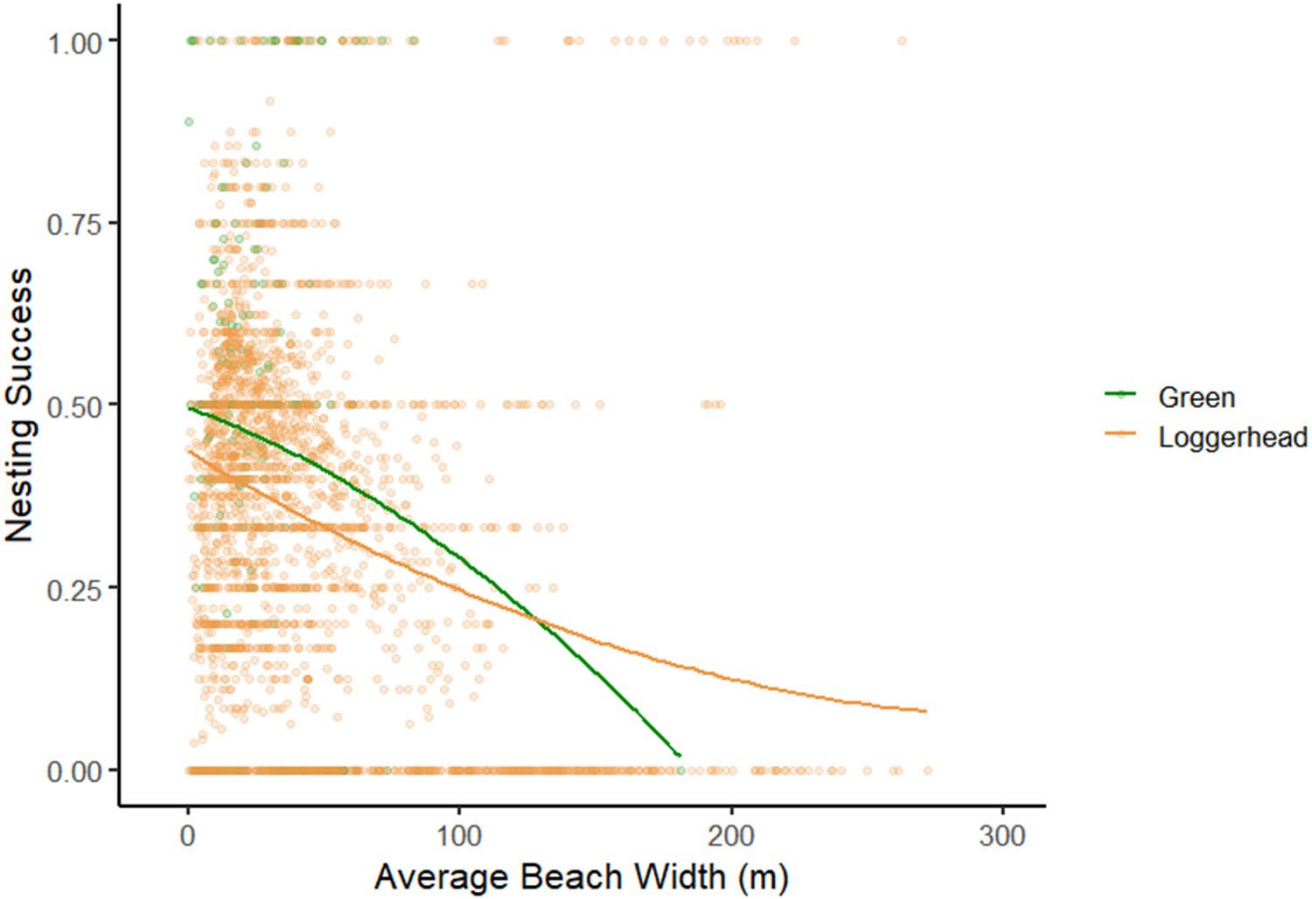
Loggerhead and green sea turtle nesting success in relation to average beach width (m). Nesting success was calculated as the proportion of sea turtle emergences that resulted in a nest. Greens rarely nested successfully on wide beaches.

For loggerheads, nesting success significantly increased from north to south (z = − 7.75, p < 0.001) and significantly decreased as the beach widened (z = − 12.84, p < 0.001). Nesting success was also significantly different when these terms interact (z = 7.28, p < 0.001). For green sea turtles: nesting success significantly decreased from north to south (z = − 2.41, p = 0.02) and significantly decreased as the beach widened (z = − 1.99, p = 0.046, Fig. 6). Emergence success for loggerheads significantly increased from north to south (z = 2.68, p = 0.007). Emergence success significantly decreased over the course of the nesting season (z = − 2.45, p = 0.014) and significantly decreased over the course of the study (z = − 2.69, p = 0.007). For green sea turtles, emergence success significantly increased over the course of the study (z = 2.45, p = 0.014); significantly increased from north to south (z = 2.05, p = 0.04), as the beach widened (z = 3.92, p < 0.001), and significantly decreased over the course of the season (z = − 2.71, p = 0.007).

For both species, nest site selection differed between the upper, middle, and lower portions of the beach (by width, Table 2). Loggerheads significantly preferred to nest in the upper portion of the beach—nearer to the dune (χ^2^ = 962.92, df = 2, p < 0.001), but they were significantly more successful at nesting in the middle (χ^2^ = 419.05, df = 2, p < 0.001). Loggerheads had a significantly higher emergence success in the upper portion of the beach (χ^2^ = 208.62, df = 2, p < 0.001), and a significantly lower incubation duration in the lower part of the beach (χ^2^ = 101.72, df = 2, p < 0.001). Green sea turtles significantly preferred to nest in the upper part of the beach near or in the dune (χ^2^ = 58.64, df = 2, p < 0.001), were significantly more successful at nesting in the upper portion (χ^2^ = 9.02, df = 2, p = 0.01), and had significantly lower emergence success in the lower portion (χ^2^ = 11.65, df = 2, p < 0.001). There was a significant difference in incubation duration in relation to location on the beach (χ^2^ = 6.06, df = 2, p = 0.048), nests in the upper portion of the beach had a higher average incubation duration.

**Table 2:** Nest site selection, nesting success, emergence success, and incubation duration within three spatial “thirds” of the beach width. Significantly different values are described in the text but are bolded in the table.

	Loggerheads				Greens			
	Nest placement	Nesting success	Emergence success	Incubation	Nest placement	Nesting success	Emergence success	Incubation
Lower	**16.7%**	**38.0%**	**40.6%**	**58.4**	**3.8%**	**38.3%**	**30.5%**	58.8
Middle	**28.2%**	**55.7%**	**48.6%**	59.6	**11.2%**	**44.7%**	**58.8%**	57.4
Upper	**55.1%**	**48.3%**	**58.2%**	59.3	**85.0%**	**51.4%**	**65.9%**	59.1

**Figure 7:**
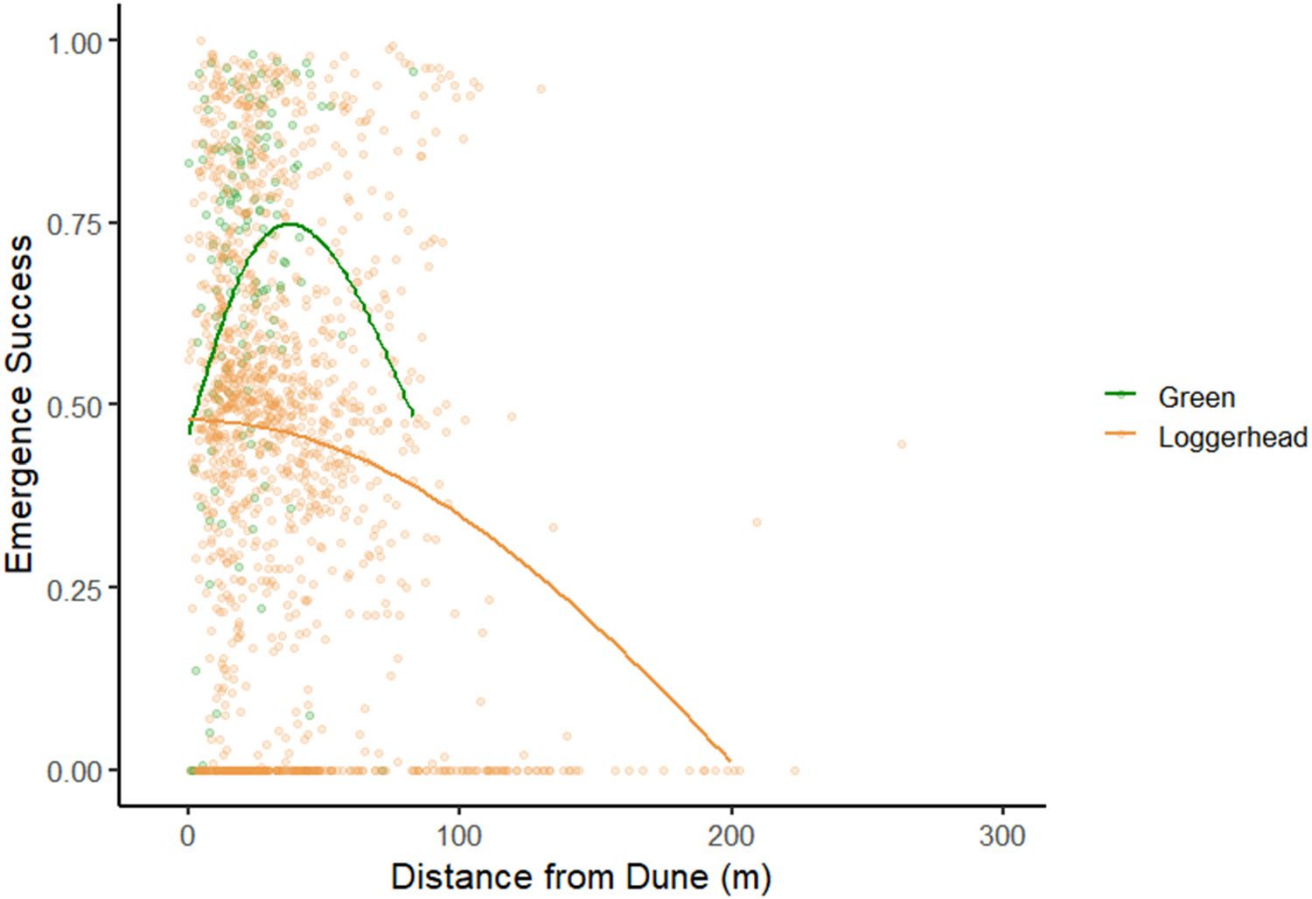
Emergence success in relation to the distance from the upper barrier (the dune). Loggerhead clutches laid closer to the dune tend to have a higher emergence rate. Few green turtles lay their clutches away from the dune and tend to have a higher success rate near, but not in the vegetation.

During some years, storm activity and high surf effectively removed the lower and middle portions of the beach over the course of the season. To account for this spatial difference, separate models were run to determine if nest placement, nesting success, emergence success, and incubation duration were impacted by the continuous variable of distance to the upper barrier or dune. For loggerheads, nest placement was significantly more likely to be closer to the upland barrier (t = − 18.77, df = 9786, p < 0.001), nesting success was significantly greater closer to the upland barrier (z = − 28.33, df = 9787, p < 0.001); emergence success significantly declined further from the upland barrier (z = − 12.72, df = 5628, p < 0.001, Fig. 7), and incubation duration significantly increased further from the upland barrier (t = 5.29, df = 4935, p < 0.001). For green sea turtles, nest placement was significantly more likely closer to the upland barrier (t = − 7.03, df = 466, p < 0.001), but nesting success did not differ (z = − 1.28, df = 466, p = 0.20); emergence success significantly declined farther from the upland barrier (z = − 1.99, df = 235, p = 0.473), and incubation duration significantly declined farther from the upland barrier (t = − 2.75, df = 223, p = 0.006).

## Discussion

Although loggerhead nesting counts have increased across Florida beaches^59^, evidence of a significant increase in the overall number of breeding females in the past 30 years is lacking^66^, hampering a strong recovery of the overall population. Few studies have looked at nesting trends on the Florida Gulf of Mexico coastline and their impact on the larger breeding population. Recent research in the Northern Gulf of Mexico suggests a reduction in the number of nests^85^. To the south on Keewaydin Island, although nest counts have increased, their hatchling production has declined^28^. Further, little information has been published about the slow recovery of green sea turtles in the Gulf of Mexico following the overharvesting of the species in the 1800’s^86,87^. However, the data presented in this paper shows that nest counts in Sarasota County have rapidly grown for both species, encouraging recovery. Beaches in Florida have partnered with FWC for their SNBS program since 1979 and the INBS program since 1989. For over 40 years, citizen scientists have provided data for the management of these beaches. Conservation efforts in this region appear to be effective and are needed.

One of the contributing factors of increased growth may be due to the implementation of turtle excluder devices (TEDs) on shrimp trawling vessels in the 1990s. Reducing bycatch invariably expands the number of sea turtles reaching sexual maturity—loggerheads reach sexual maturity between 27 and 38 years^13,24^ and green sea turtles reach sexual maturity between 19 and 44 years^22,88,89^. Thus increasing the number of potential nesters for future generations. There was an estimated 60% reduction in bycatch of all species of sea turtles in the Atlantic following the TED regulation (mortality: 94% reduction^90^). The usage of TEDs and the increased awareness in the region have provided 1–2 generations worth of potential nesters to aid in recruitment and growth of the population. Additionally, these increased recruitment rates could be due to ocean currents in the Gulf of Mexico.

Recruitment to sea turtle nesting aggregations is strongly influenced by proximity to the Gulf Stream System (GSS), which affects overall population structure^91^. Putman and colleagues modelled that the GSS accounted for 90% of the spatial variation in regional nesting density on the Atlantic and may explain why rookeries on the east coast of Florida will continue to dramatically increase in recruitment. In the Gulf of Mexico, the Loop Current passes near the western coast of Florida before joining the GSS^92^, and may explain increased nesting in the Sarasota County region in relation to the rest of the Florida rookeries on the Gulf of Mexico. From 2016 to 2021, loggerhead nesting counts in Sarasota County (which includes beaches monitored by MML STCRP and the Coastal Wildlife Club) accounted for 44.4% of all nesting on Florida beaches on the Gulf of Mexico and 50.1% for greens^59,60^. Genetically, the loggerhead population nesting in the region is distinct from management units to the north (St. Georges Island, Florida) and to the south (Keewaydin Island, Florida)^93^ and the central west region is one of the fastest growing nesting populations in Florida^66^. While biogeography may help explain the overall population shifts, individual assessments could explain the finer differences by beach.

Loggerheads and green sea turtles prefer to nest on beaches with less artificial light and on shorelines that are less developed^94–96^. Comparable to previous observations^87,97^, both species in the Sarasota region also prefer to nest closer to the upland barrier. Nesting success has significantly declined over the course of the study, likely due to increased coastal housing density and construction near the beach^49,98^. Increases in coastal development result in more obstructions on the beach deterring nesters^71,99^, more lighting issues that discourage nesters^45,100^, and more nourishment or beach armoring that can prevent nesters from approaching the beach^43,65,101^. Two of the beaches with the lowest nesting success and nesting density (Lido Key, and Longboat Key) are also the beaches that have been (re)nourished the most in the county. Casey Key is the least developed and it has the highest nesting density. Unfortunately, modeling of nesting beach inundation into the future suggests that sea level rise could cover 5–32% of suitable nesting beach widths by the year 2100^102–104^. A recent report from NOAA estimates that by 2050 sea levels will rise on average between 0.25 and 0.30 m, increasing the likelihood of coastal flooding, storm surge impact, and coastal erosion^105^. Nesting density has continued to increase in line with the nest counts, which could be an exacerbating factor if the suitable nesting areas decline due to sea level rise and coastal development.

Nesting females may increase their fitness by laying clutches earlier in the season, in varied locations over the course of the season, and by varying the number of eggs per clutch^106^. Loggerhead nests laid later in the season had a much lower emergence success rate—likely due to the increased level of tropical storms and predation over the course of the season. Regional temperature increases at foraging areas are influencing sea turtle nesting phenology^107–109^. Although longer-lived females consistently lay more clutches over the course of the season^110^, few estimates of true nest counts derived from satellite tagging data have been published^15,111–113^ and updates are needed. Further modeling of how clutch frequency and clutch size has changed over time in relation to rising temperature and sea levels should be prioritized for nesting beaches on the Gulf of Mexico.

Throughout Florida, air temperature rises over the course of the nesting season, but different abiotic factors influence how nest temperatures fluctuate. Increased temperature influences embryo mortality^114^, but nests in the Sarasota region typically get more rainfall than nests on the east coast of Florida at similar latitudes and thus tend to be cooler (Lolavar & Wyneken *pers. comm*). The six beaches have different sand particle size and reflectance (albedo) that affect incubation temperature and duration (e.g., nest incubation duration is shorter on the dark, coarse sand of Venice than on the white, silty sand of north Siesta). An assessment is needed for the region to determine how physical characteristics (sand type, slope, albedo, etc.) influence nest temperatures. Green turtles tend to dig deeper egg chambers, that are placed closer to the upper barrier resulting in few green turtle nests that fully washout (barring a hurricane). On the Gulf coast of Florida, green sea turtles are considered to be a rare species and most of their nests were caged to prevent depredation and increase potential recruitment. Previous caging protocols are a likely factor of the discrepancy in emergence success between the two species^115^. However, the green nesting population is recovering and since 2021, MML STCRP has stopped caging all green nests.

Following emergence, 6–7.6% of hatchlings do not make it to the water^77,116^, thus reducing potential recruitment. Sky glow and artificial lighting sources lead hatchlings towards the dune and increase the likelihood of depredation or exhaustion. From 2018 to 2021 misorientation/disorientation events in Sarasota County made up 9% of all recorded events in the state of Florida^117^. Models from Mazaris and colleagues identified that increasing hatchling recruitment even at a single site, would increase the overall hatchling production from entire nesting aggregations^118^. Future projects should assess lighting issues in relation to disorientation events and hatchling mortality in the Sarasota region.

The data presented here documented the expanding nesting population for a productive Gulf of Mexico rookery. Although total nesting counts in the Gulf of Mexico annually accounts for only 7% of all loggerhead nesting in Florida, these beaches have a significant hatchling production annually reinforcing recovery. Primary, adult, and breeding sex ratios should be assessed to quantify the population age structure and determine if there is any risk of a sex ratio skew that other rookeries are experiencing^119,120^. Projects should model how ocean level increases will decrease suitable nesting habitat and how coastal development exacerbates the issue^103,105^. When suitable nesting area decreases, will there be an effect on carrying capacity or will nesters migrate to less suitable beaches? Recruitment is critical to lasting recovery, and protection measures are needed to maintain population growth.

### Supplementary Information

Supplementary Information.

## Data Availability

The data sets generated during and/or analyzed during the current study will be available by request. Please contact the corresponding author at jlasala@mote.org for information.
