# Supplementary material for: Forty years of monitoring increasing sea turtle relative abundance in the Gulf of Mexico
Source: Sci Rep. 2023 Oct 11;13:17213. doi: 10.1038/s41598-023-43651-4 (PMC10567714; doi:10.1038/s41598-023-43651-4)
Supplement: Supplementary file 1 — Supplementary Information. [file 41598_2023_43651_MOESM1_ESM.docx]

**Supplementary Information**

1. **Full data set.**
2. Estimated hatchling production by beach from 1982-2021.

|  | Longboat Key | | Lido Key | | North Siesta Key | | South Siesta Key | | Casey Key | | Venice | |  | |
| --- | --- | --- | --- | --- | --- | --- | --- | --- | --- | --- | --- | --- | --- | --- |
| Year | Actual | Most Likely | Actual | Most Likely | Actual | Most Likely | Actual | Most Likely | Actual | Most Likely | Actual | Most Likely |  |  |
| **1982** | 414 | | 0 | | 0 | | 451 | | 5131 | | 828 | |  |  |
| **1983** | 1040 | | 68 | | 93 | | 1709 | | 11028 | | 682 | |  |  |
| **1984** | 2529 | | 72 | | 566 | | 1984 | | 7927 | | 536 | |  |  |
| **1985** | 3718 | | 66 | | 236 | | 1411 | | 3827 | | 521 | |  |  |
| **1986** | 6796 | | 248 | | 329 | | 5547 | | 24006 | | 4031 | |  |  |
| **1987** | 7339 | | 188 | | 249 | | 4081 | | 18820 | | 3039 | |  |  |
| **1988** | 7973 | | 158 | | 72 | | 2526 | | 13691 | | 3091 | |  |  |
| **1989** | 12375 | | 342 | | 704 | | 5840 | | 28758 | | 4416 | |  |  |
| **1990** | 3344 | | 785 | | 896 | | 6855 | | 34996 | | 4399 | |  |  |
| **1991** | 11687 | | 1579 | | 2079 | | 9184 | | 36947 | | 6603 | |  |  |
| **1992** | 8575 | | 2088 | | 1791 | | 6586 | | 21085 | | 6585 | |  |  |
| **1993** | 10756 | | 2280 | | 1345 | | 9401 | | 39041 | | 8598 | |  |  |
| **1994** | 14811 | | 2410 | | 2339 | | 11175 | | 41336 | | 11926 | |  |  |
| **1995** | 14132 | | 2153 | | 554 | | 11567 | | 47536 | | 10331 | |  |  |
| **1996** | 19140 | | 3859 | | 746 | | 12418 | | 45530 | | 19104 | |  |  |
| **1997** | 18195 | | 3522 | | 1119 | | 14043 | | 42343 | | 16968 | |  |  |
| **1998** | 13258 | | 1781 | | 914 | | 12169 | | 42018 | | 14349 | |  |  |
| **1999** | 22448 | | 1788 | | 1414 | | 15453 | | 40431 | | 19080 | |  |  |
| **2000** | 21010 | | 2653 | | 1507 | | 13226 | | 30670 | | 16035 | |  |  |
| **2001** | 8901 | | 423 | | 331 | | 4920 | | 13765 | | 10929 | |  |  |
| **2002** | 15036 | | 1651 | | 601 | | 9282 | | 23639 | | 12026 | |  |  |
| **2003** | 17696 | | 1060 | | 612 | | 13353 | | 35884 | | 14441 | |  |  |
| **2004** | 7933 | | 1328 | | 91 | | 3300 | | 18554 | | 9061 | |  |  |
| **2005** | 2568 | | 832 | | 331 | | 3344 | | 17365 | | 4857 | |  |  |
| **2006** | 10309 | | 2036 | | 332 | | 6971 | | 25751 | | 10702 | |  |  |
| **2007** | 8227 | | 1739 | | 636 | | 3529 | | 15510 | | 6181 | |  |  |
| **2008** | 10849 | | 1530 | | 175 | | 7839 | | 23843 | | 11173 | |  |  |
| **2009** | 13240 | | 1147 | | 614 | | 4461 | | 24157 | | 9346 | |  |  |
| **2010** | 12230 | | 608 | | 723 | | 4830 | | 31715 | | 10997 | |  |  |
| **2011** | 14918 | | 2099 | | 1111 | | 4641 | | 26732 | | 12598 | |  |  |
| **2012** | 21471 | | 2417 | | 1909 | | 8076 | | 19558 | | 14030 | |  |  |
| **2013** | 13960 | 29587.7 | 809 | 3173.9 | 218 | 1973.4 | 1991 | 13545.2 | 9724 | 37805.2 | 2814 | 14461.2 |  |  |
| **2014** | 11766 | 25664.7 | 6157  3301  4267  4465  3093  1948  5247  4937 | | 2447  806  1368  3350  2540  1086  2884  1823 | | 1168 | 10318.0 | 15619 | 48504.6 | 2482 | 14791.5 |  |  |
| **2015** | 12137 | 30865.2 |  |  |  |  | 1995 | 15916.2 | 8566 | 38658.3 | 7821 | 12013.7 |  |  |
| **2016** | 27616 | 53123.0 |  |  |  |  | 2856 | 18562.4 | 13159 | 80707.8 | 4925 | 31273.1 |  |  |
| **2017** | 24809 | 55538.8 |  |  |  |  | 9134 | 25340.0 | 20215 | 73272.4 | 4870 | 26563.3 |  |  |
| **2018** | 27756 | 44584.8 |  |  |  |  | 4967 | 15780.9 | 13458 | 50245.7 | 6549 | 23702.2 |  |  |
| **2019** | 31216 | 57602.4 |  |  |  |  | 7612 | 29453.7 | 23728 | 86355.0 | 3973 | 30556.7 |  |  |
| **2020** | 6590 | 49747.2 |  |  |  |  | 1873 | 17868.7 | 21969 | 60186.2 | 2455 | 23670.8 |  |  |
| **2021** | 25048 | 45950.3 |  |  |  |  | 6071 | 23108.3 | 21709 | 57776.7 | 7877 | 31366.6 |  |  |
|  |  |  |  |  |  |  |  |  |  |  |  |  |  |  |
| **Sum** | 523816 | 735582.2 | 77134 | 79498.9 | 40941 | 42696.4 | 257839 | 390065.5 | 959741 | 1345105.8 | 321229 | 485862.2 |  |  |
|  |  |  |  |  |  |  |  |  |  |  |  |  |  |  |

Known 2,180,700

Most- Likely 3,078,811

**Table A1:** From 1982-2012, MML STCRP excavated all nests and hatchling productivity could be quantified completely. Starting in 2013, different beaches received different survey “treatments” that are described in detail in the methods. All beaches had at least one day that all nests were marked and subsequently monitored. Nesting trends tend to differ by beach and attempts to quantify overall hatchling production based on these monitoring methods resulted in high error. Instead, we ran multiple regression analyses to model hatchling production by beach from 1982-2012 (independent variables: total nest number, average number of eggs per nest; dependent variable: number of hatchlings produced) and then used the resulting coefficients to estimate how many hatchlings could have emerged from their nests from 2013-2021. Average emergence success values were not included in this model to reduce potential bias from extreme weather events that can affect year-year variation. The known number of hatchlings and the most likely number of hatchlings by beach based on 95% CI are presented in this table.

2. Nesting density (nests per km), emergence success and incubation duration by beach

|  | Loggerhead | | | Green | | |
| --- | --- | --- | --- | --- | --- | --- |
|  | Nesting Density | Emergence Success | Incubation Duration | Nesting Density | Emergence Success | Incubation Duration |
| Longboat Key | 19.79 | 52.4% | 58.3 | 0.29 | 67.1% | 59.5 |
| Lido Key | 9.80 | 45.3% | 62.3 | 0.19 | 47.9% | 68.0 |
| North Siesta Key | 7.19 | 27.1% | 71.6 |  |  |  |
| South Siesta Key | 48.76 | 47.7% | 57.9 | 0.90 | 55.4% | 56.1 |
| Casey Key | 62.09 | 57.7% | 59.7 | 1.56 | 66.9% | 60.3 |
| Venice | 36.87 | 47.5% | 55 | 0.18 | 71.0% | 54.3 |

**Table A2**: Loggerhead and green sea turtle nesting density, emergence success and incubation duration by beach. North Siesta Key has yet to have a green nest. Each beach is unique and contributes to the overall nesting aggregation in the region.

1. Best fitting negative binomial generalized linear model of nesting success

Nesting Success ~ Year*JND+FDEP*BW

|  | Standard Error | z value | p value |
| --- | --- | --- | --- |
| Year | 0.0051 | -1.705 | 0.088 |
| Julian Nest Date (JND) | 0.0224 | 0.112 | 0.911 |
| FDEP Monument | 0.0292 | -7.754 | <0.001* |
| Beach Width (BW) | 0.0320 | -12.844 | <0.001* |
| Year : JND | 0.0025 | -0.123 | 0.902 |
| FDEP : BW | 0.0067 | 7.279 | <0.001* |

**Table A3:** The best fitting GLM for nesting success of loggerhead sea turtles. Dates of nest deposition (by year or day) were not significant factors of nesting success. However, where a nest was placed (using the FDEP regional marker as a proxy for location), beach width, and the interaction of these two variables were highly significant factors.

1. **Nourished Beaches Removed by Year**

Few beaches in Florida have not been nourished in the past 60 years. Sarasota County beaches were nourished multiple times throughout the 40-year history of MML STCRP monitoring. Many projects were completed prior to nesting season, but turtle nesting behavior can differ due to nourishment.^43^ The full data set in the paper included the nest counts and the false crawl counts of all beaches regardless of nourished status to calculate the nesting success, nesting density, beach width of each beach and year, and nest site selection. All nests that were relocated (including for nourishment) were not included in the full data set analyses of emergence success, incubation, or hatchling production. Relocated nests can differ in success to *in situ* nests^43, 120^ and this step was included to remove potential bias.

However, to potentially remove any further bias from nourishment areas, we include supplementary analyses here that remove the beach by year that nourishment was completed. Data were removed regardless if the nourishment was completed prior to the nesting season of the same year or during the nesting season. Full beaches were removed instead of the specific regions impacted by the nourishment because turtle behavior could be influenced by newly nourished sand to nest elsewhere on the beach^63^ - influencing nesting numbers in those un-nourished areas. This removal impacts 23 years across four beaches (Longboat Key, Lido Key, South Siesta Key, and Venice), culminating in the removal of 12,893 nests and 16,298 false crawls of four species (loggerhead, green, leatherback, and Kemp’s ridley; Table B1).

1. Beach nourishment completion years and nest counts by beach removed from the supplementary model (Loggerhead, Green, Leatherback, Kemp’s Ridley)

| Longboat Key | | Lido Key | | South Siesta Key | | Venice | |
| --- | --- | --- | --- | --- | --- | --- | --- |
| Year | # Nests | Year | # Nests | Year | # Nests | Year | # Nests |
| 1982 | 37, 0, 0,0 | 1985 | 6, 0, 0, 0 | 2006 | 109, 0, 0, 0 | 1994 | 187, 1, 0, 0 |
| 1991 | 157, 0, 0, 0 | 1991 | 23, 0, 0, 0 | 2016 | 359, 0, 0, 0 | 1996 | 263, 0, 0, 0 |
| 1993 | 132, 0, 0, 0 | 1996 | 50, 0, 0, 0 |  |  | 2005 | 195, 0, 0, 0 |
| 1996 | 288, 0, 0, 0 | 1998 | 42, 0, 0, 0 |  |  | 2015 | 255, 1, 0, 0 |
| 1997 | 260, 0, 0, 0 | 2001 | 16, 0, 0, 0 |  |  | 2019 | 643, 2, 1, 1 |
| 2001 | 261, 0, 1, 0 | 2003 | 32, 0, 0, 0 |  |  |  |  |
| 2003 | 293, 1, 0, 0 | 2009 | 17, 0, 0, 0 |  |  |  |  |
| 2006 | 160, 0, 0, 0 | 2015 | 97, 0, 0, 0 |  |  |  |  |
| 2010 | 270, 1, 0, 0 | 2019 | 96, 2, 0, 0 |  |  |  |  |
| 2011 | 275, 0, 0, 0 | 2020 | 153, 0, 0, 0 |  |  |  |  |
| 2014 | 543, 1, 0, 0 |  |  |  |  |  |  |
| 2015 | 693, 5, 0, 0 |  |  |  |  |  |  |
| 2016 | 1184, 0, 0, 0 |  |  |  |  |  |  |
| 2017 | 1260, 17, 0, 0 |  |  |  |  |  |  |
| 2018 | 993, 0, 0, 0 |  |  |  |  |  |  |
| 2019 | 1309, 33, 0, 0 |  |  |  |  |  |  |
| 2020 | 1123, 13, 0, 0 |  |  |  |  |  |  |
| 2021 | 1026, 6, 0, 0 |  |  |  |  |  |  |

**Table B1**: This table describes the total number of nests by species removed from the supplementary model that were impacted by nourishment across the study by beach and year. Many nourishment projects are piecewise across the beaches and to account for that potential bias all nests from those beaches were removed from the model.

Supplementary analyses for both loggerheads and greens include nest counts, and the comparison of nesting success, nesting density, emergence success, and incubation duration in the new model to the original full data set. Beach width and nest site selection are not reassessed because nourished sand gradually declines over time (as do natural beaches), but the rate of decline was not assessed during the 40-year study. Future projects should assess this decline in relation to nourished and un-nourished beaches on other long-term nesting projects.

Loggerhead and green nest counts were not normal (Shapiro-Wilk: W = 0.680, p < 0.001; W =0.442, p < 0.001 respectively) and neither were their residuals. Kruskal-Wallis tests were run to determine if there was a significant difference in nest counts due to year and beach. For loggerheads, there was a significant difference in nest counts due to year (χ^2^ = 65.186, df = 39, p = 0.005) and due to beach (χ^2^ = 123.28, df = 5, p<0.001). These results are similar to the full model and post hoc tests (Dunn) result in similar findings – nest counts are increasing over time and Casey Key has more nests than the other beaches, Lido and North Siesta Key have the least number of nests. For green sea turtles, there was not a significant difference in nest counts due to year (χ^2^ = 19.804, df = 22, p = 0.595), but there was a significant difference in nest counts in relation to beach (χ^2^ = 13.689, df = 4, p = 0.008). Green nest counts are increasing, but not significantly and greens prefer to nest on Casey Key, then South Siesta Key and have never nested on North Siesta Key.

1. Loggerhead and green sea turtle average nesting success, nest density, emergence success, and incubation duration with nourished beaches by year removed

|  | Loggerhead | | | | Green | | | |
| --- | --- | --- | --- | --- | --- | --- | --- | --- |
|  | Nesting Success | Nesting Density | Emergence Success | Incubation Duration | Nesting Success | Nesting Density | Emergence Success | Incubation Duration |
| Longboat Key | 0.49 | 11.08 | 53.3% | 58.7 | 0.54 | **0.10** | 43.2% | 59 |
| Lido Key | 0.50 | 9.81 | 44.7% | 62 | 0.25 | 0.19 | 95.9% | 66 |
| North Siesta Key | 0.38 | 7.19 | 27.1% | 71.6 |  |  |  |  |
| South Siesta Key | 0.58 | 48.14 | 48.1% | 57.9 | 0.52 | **1.09** | 55.4% | 56.1 |
| Casey Key | 0.56 | 62.09 | 57.7% | 59.7 | 0.53 | 1.70 | 66.9% | 60.3 |
| Venice | 0.51 | 32.19 | 47.8% | 55 | 0.62 | 0.22 | 65.1% | 53.8 |

**Table B2:** This table shows the overall trends of loggerhead and green nests if nourished beaches are removed during the year of nourishment. The two data sets were not significantly different for any loggerhead and most green sea turtle parameters. However, nesting density for green sea turtles was lower on Longboat Key and higher on South Siesta in this model **(bolded).**

Data from the full data set and the nourishment removed data set were pooled to test for normality. None of the variables (nesting success, nesting density, emergence success, and incubation duration) were normal for either species (Shapiro-Wilk: Loggerhead – W = 0.969, p < 0.001; W = 0.795, p < 0.001; W = 0.975, p < 0.001; W = 0.917, p < 0.001; Green –W = 0.909, p < 0.001; W = 0.513, p < 0.001; W = 0.909, p < 0.001; W = 0.948, p = 0.001 respectively) and none of the residuals were normal. All analyses were non-parametric. Mann Whitney U tests were run to determine if there was a statistically significant difference between the full data set and the nourishment removed data set for these four variables.

For loggerheads, there were no significant differences in nesting success, nesting density, emergence success, or incubation duration between the two data sets (W = 23266, p = 0.368; W = 24408, p = 0.742; W = 26089, p = 0.163; W = 21615, p = 0.728 respectively). For greens, there were no significant differences in nesting success, emergence success, and incubation duration (W = 2237.5, p = 0.805; W = 1160.5, p = 0.847; W = 1191, p = 0.588 respectively), but there was a significant difference in nesting density (W = 1406, p = 0.035). Of the four beaches with nourishment events, the nesting density of greens per km fell on Longboat Key and rose on South Siesta, although as greens did not nest on South Siesta Key the two seasons that were removed– this is more likely due to monitoring variation than differing sea turtle behavior.
